# Effect of Holding Time on the Extrusion Force and Microstructure Evolution during the Plastic Forming of Ti-6Al-4V Micro-Gears

**DOI:** 10.3390/ma15041507

**Published:** 2022-02-17

**Authors:** Xiangzhong Yan, Shengwei Zhang, Kunlan Huang, Yi Yang, Wei Wang, Mingxia Wu

**Affiliations:** 1 School of Mechanical Engineering, Sichuan University, Chengdu 610065, China; lutxiang@126.com (X.Y.); yangyi@scu.edu.cn (Y.Y.); 2019223025065@scu.edu.cn (W.W.); wumingxia@scu.edu.cn (M.W.); 2 AECC AERO Science and Technology Co., Ltd., Chengdu 610503, China; zswabc1111@163.com

**Keywords:** Ti-6Al-4V alloy, micro-gear, plastic deformation, extrusion force, microstructure evolution, reconstructed β grains

## Abstract

The application of titanium alloy micro-gears in microelectromechanical systems has been severely restricted, as the graphite mold is prone to abrasion or even to crack at high temperatures, mainly due to the forming load. We aimed to manufacture Ti-6Al-4V alloy micro-gears through hot extrusion under an electric field and to clarify the influence of holding time on the extrusion force. The results suggest that the formed gears had a complete filling and clear tooth profile. Moreover, the contact resistance and current density caused a gradient temperature distribution inside the billet, resulting in a carburized layer and inhomogeneous β grains. The extrusion force increased with an increased holding time, which can be ascribed to the increase in the thickness of the carburized layer and the β grain size. Among these two factors, β grain size played a leading role in the extrusion force. Continuous dynamic recrystallization dominated the deformation in a single β phase, and the misorientation of the transformed α laths from β grains followed the Burgers orientation relationship. This study may pave the way for the extrusion forming of other titanium alloy micro-components.

## 1. Introduction

Micro-gears are pivotal actuating elements in the transmission of power and motion and are used extensively in microelectromechanical systems (MEMS), including medical treatment, aviation, automobile, communication, and military applications [1]. Some forming methods and materials of micro-gears are listed in Table 1. It has been shown that extrusion is the most advantageous of these methods due to its high efficiency, high-volume throughput, and low unit cost. Titanium (Ti) alloys have excellent properties, such as low densities, excellent corrosion resistance, high strength-to-weight ratios, excellent fatigue performance, and biocompatibility [2]. Li et al. [3] simulated the hot extrusion of Ti–6Al–4V alloy and the results were compared with those obtained experimentally. Ye et al. [4] investigated the dynamic recrystallization and strengthening-toughening effects in a near-α Ti-xSi alloy processed by hot extrusion. Yang et al. [5] studied the microstructure evolution and mechanical properties of P/M Ti-22Al-25Nb alloy during hot extrusion. Currently, there is much research on the macro-extrusion of titanium alloys, while there is little literature on the extrusion formation of micro-gears using difficult-to-deform Ti alloys, due to high strength, poor plasticity, and the mold’s vulnerability. These properties dictate that Ti alloy micro-gears should be formed at high temperatures.

In terms of hot extrusion, H13 steel molds were used to extrude wide thin-ribbed sections at a temperature of 480 °C [6]. The aluminum alloy tubing was extruded at the molds’ face temperatures of 460–490 °C and the molds were made from H13 [7]. Li et al. [8] studied the effects of the asymmetric feeder on the microstructure and mechanical properties of Al-Zn-Mg alloy by hot extrusion using H13 molds at a temperature of 500 °C. Liu et al. [9] also used H13 molds to extrude AZ31 magnesium alloy at 370 °C. Molds are generally manufactured using H13 hot work tool steel, at an extrusion temperature that is no more than 500 °C. When the surface temperature of a mold exceeds 500 °C, the mold’s hardness is reduced and the external force can easily cause plastic deformation as well as abrasive wear of the mold material [10,11]. Of the two, plastic deformation is the main factor of mold failure [11]. Our previous research in the die forging of Ti-6Al-4V micro-gears showed that the optimal forming temperature was 1200 °C [12]. This temperature can cause metal molds to suffer severe plastic deformation, which further contributes to the inferior geometry of the extrudates, and possibly extrusion failure. In contrast, graphite molds have excellent thermal and electrical conductivity, a low linear expansion coefficient, thermal stability, and a good lubricity [13]. However, the graphite molds are prone to brittle fracture under an external load, although the strength of graphite increases with increased temperature, especially at high temperatures.

Elevated temperatures were shown to contribute to the decline in the necessary extrusion force [14]. In Ti alloy, the α grains can transform into β grains and then grow quickly at temperatures above the phase transformation temperature. Grain size has a considerable impact on the deformation behaviors and mechanical responses of materials in the micro-forming process. Zheng et al. [15] and Fu et al. [16] demonstrated that flow stress was decreased with an increase in the pure copper grain size through uniaxial tensile tests. The research by Zhang et al. [17], showed that fine grains of austenite SS 316L metallic sheet with a thickness of 100.0 μm had higher yield stress of 260.5 MPa than coarse grains of 196.5 MPa by the monotonic uniaxial tensile tests. Zheng et al. [18] discovered that the forming pressure decreased with the increased grain size of pure copper in the progressive forming process. The coarse grains facilitated the forming of desirable geometries and reduced dimples on the shearing surface. All the experiments mentioned above were carried out at room temperature. Until now, the relationship between grain sizes and flow stress or forming pressure was widely conducted for superior plasticity materials. As such, there is a lack of research on the relationship between grain sizes and flow stress or forming pressure for hard-to-deform Ti alloy at elevated temperatures.

Therefore, in this study, the Ti-6Al-4V micro-gears were manufactured by hot extrusion with graphite molds, with a module of 0.3 mm and eight teeth. The tooth depth was 0.675 mm and the tooth thickness was 0.471 mm. The different grain sizes were obtained through different holding times, to investigate the influence of grain size on the extrusion force during the hot extrusion process.

## 2. Materials and Methods

### 2.1. Materials

**Figure 1:**
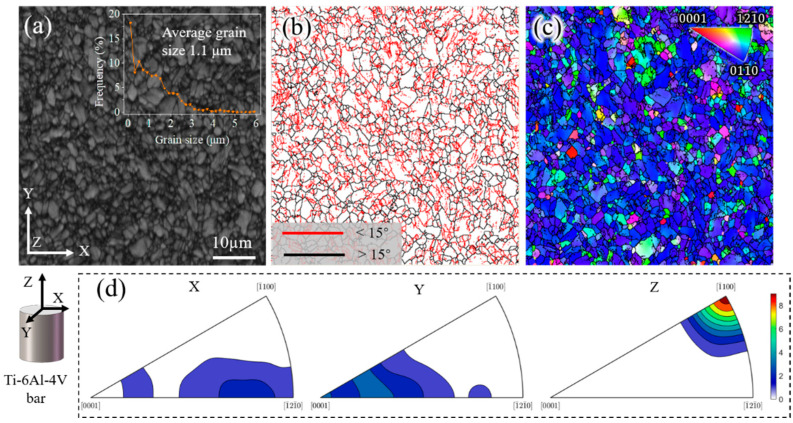
Microstructure of the as-received Ti-6Al-4V billet bar. (**a**) Band contrast and grain size; (**b**) grain boundaries map, where the grain boundaries colored red are <15° and black are >15°; (**c**) inverse pole figure map parallel to Z; and (**d**) inverse pole figures parallel to X, Y, and Z.

The as-received commercial Ti-6Al-4V alloy (From Shanghai Yingxiong Metal Material Co., Ltd., Shanghai, China) was a hot-drawn bar, with a chemical composition (in wt%) of 6Al-3.9V-0.15O-0.008H-0.02C-0.03N-0.12Fe. According to the characterization results obtained using electron back-scattered diffraction (EBSD), a near-equiaxed grain structure with an average grain size of 1.1 μm and 78.8% low-angle grain boundaries (LAGBs) in the Ti-6Al-4V billet was observed, as shown in Figure 1a,b. The LAGBs indicate that the billet had high storage energy, favoring recrystallization nucleation. The inverse pole figures in Figure 1c,d show a strong texture component of <-1100> parallel to the bar Z-direction owing to the drawing characteristics. Ti-6Al-4V is a two-phase (α + β) alloy composed of about 95% α phase at room temperature. The α grains in the initial, room temperature Ti-6Al-4V alloy, transform into β grains, which then grow quickly above the β transformation temperature of around 975 °C [27].

### 2.2. Methods

**Figure 2:**
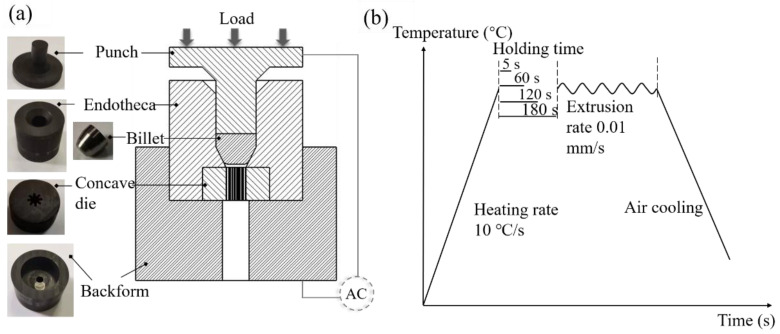
(**a**) Schematic diagram of forming Ti-6Al-4V micro-gear; (**b**) Processing route.

Figure 2a shows the extrusion diagram and graphite molds, including punch, endotheca, concave die, and backform. Figure 2b displays the extrusion process. The molds and billet were heated to the extrusion temperature of 1200 °C in a vacuum chamber using a Gleeble-1500D thermal-mechanical simulator at a heating rate of 10 °C/s and were then held for 5 s, 60 s, 120 s, or 180 s under a steady clamping force of 30 Kgf. Then, the billets were extruded at a rate of 0.01 mm/s, followed by air cooling. The corresponding parameters are listed in Table 2.

### 2.3. Characterization

The microstructural features of the billet after the assigned holding time were examined using different characterization techniques, such as optical microscopy (OM, ZEISS AxioCam MRC5, Göttingen, Germany) and a scanning electron microscope (SEM, Helios G4 UC, Thermo Fisher Scientific, Waltham, MA, USA). For OM, the specimens were mechanically polished with SiC grinding paper up to a fine 5000 grit size and were then etched in Kroll’s reagent (i.e., 2% HF, 6% HNO_3_, and the balance H_2_O). The changes in C content were conducted by energy dispersive spectrometry (EDS, Helios G4 UC) line-scanning. Elemental quantification was performed on an EPMA-1720H electron probe microanalyzer (EPMA) equipped with a wavelength dispersive X-ray spectrometer (WDS). An FEI Quanta 650F instrument equipped with HKL channel 5 analysis software (HKL Technology, Hobro, Denmark) was used for electron backscatter diffraction (EBSD) measurement of the original and remaining billets. The scanning areas of the original and remaining billets were 50×50 and 500×1000 μm^2^ with a scanning step size of 0.1 μm and 1 μm, respectively, for microstructure analysis. According to the Burgers orientation relationship, the prior β grains of the remaining billet were reconstructed employing EBSD data using the MATLAB toolbox MTEX 5.7.0. The temperature distribution of the billet was simulated by COMSOL Multiphysics 5.4 software (COMSOL, Inc., Stockholm, Sweden). A new infrared thermometer detected the actual temperature (LumaSense Technologies, Inc., Santa Clara, CA, USA).

## 3. Results and Discussion

### 3.1. Formed Micro-Gear and Extrusion Force of Different Holding Times

**Figure 3:**
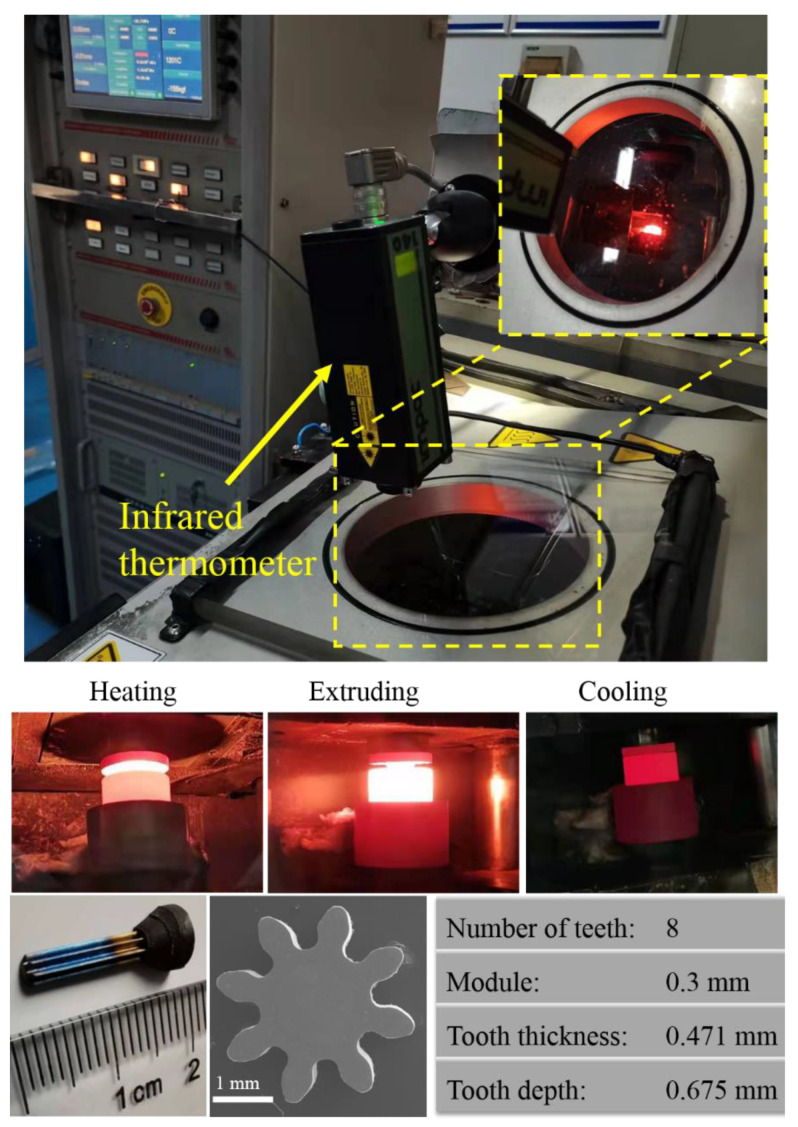
The forming process and the formed Ti-6Al-4V micro-gear.

**Figure 4:**
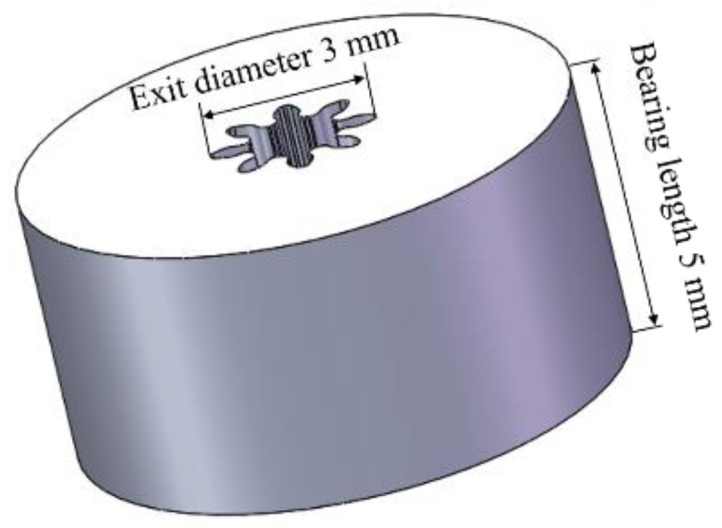
The bearing length and exit diameter of the concave die.

The Ti-6Al-4V micro-gears can be successfully fabricated by hot extrusion under thermal-mechanical coupled fields. As Figure 3 shows, the micro-gear had the following key characteristics: eight teeth, a module of 0.3 mm, a tooth thickness of 0.471 mm, and a tooth depth of 0.675 mm. The tooth profile of the formed micro-gear was completely filled and clear, demonstrating that applying the technique to manufacture Ti-6Al-4V micro-gears is feasible. Additionally, the curvature in the micro-extrusion significantly affected the final extrudates’ geometry, which was caused by the grain size, grain boundary, grain orientation, and bearing length. For all conditions, the curvature of the micro-parts could be eliminated when the bearing length was greater than the exit diameter [28]. The extruded micro-gear rod was straightened in the experiments because the bearing length was approximately 1.6 times the exit diameter (Figure 4).

**Figure 5:**
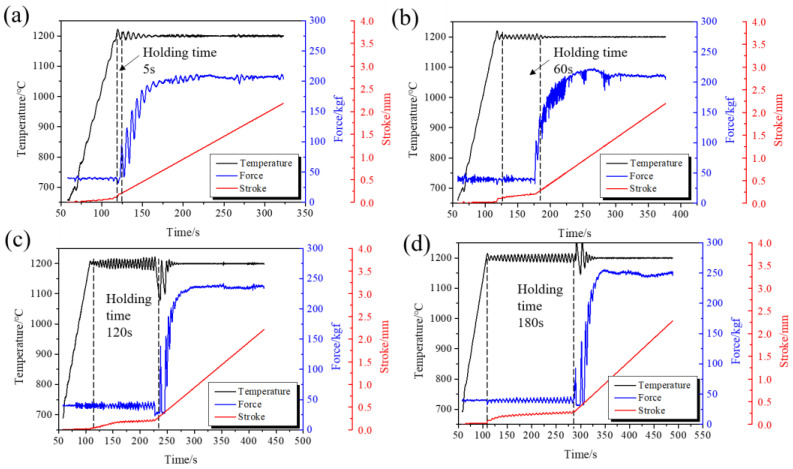
Extrusion force, temperature, and stroke displacement curves during the hot extrusion at different holding times. (**a**) 5 s; (**b**) 60 s; (**c**) 120 s; and (**d**) 180 s.

The extrusion force is one of the crucial parameters during extrusion. An excessive extrusion force results in the severe abrasion of the graphite molds, which affects the dimensional accuracy of the extruded products and increases costs, possibly even damaging the molds. So the forming load must be controlled. As shown in Figure 5, the extrusion force increased with extended holding times, indicating that a short holding time is beneficial to extrusion forming. The punch had a certain stroke displacement during the holding time due to creep.

### 3.2. The Effect of Holding Time on the Microstructure Evolution of Billets

**Figure 6:**
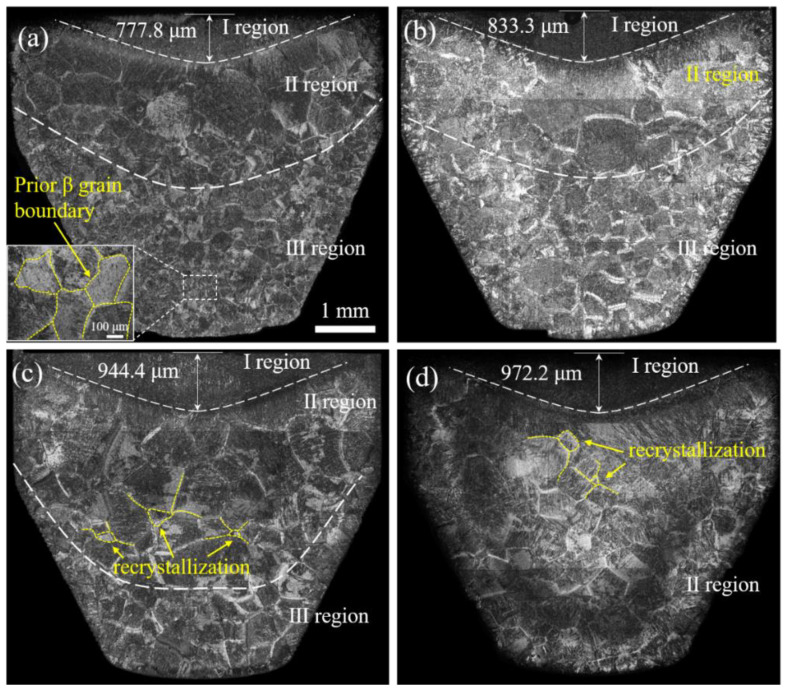
The billet microstructure at the end of each holding time. (**a**) 5 s; (**b**) 60 s; (**c**) 120 s; and (**d**) 180 s.

The holding time caused differences in the microstructure which affected the extrusion force further. In order to understand the influence of the microstructure characteristics on the extrusion force, Figure 6 shows the billet microstructure at the end of each holding time under a steady clamping force of 30 Kgf. Interestingly, the billet showed three different microstructures. The prior β grain sizes in the Ⅱ region were larger than that of the Ⅲ region when holding for 5 s, 60 s, and 120 s. By contrast, there were only two kinds of microstructures after holding for 180 s; the grain distribution in the Ⅱ region was uniform beside a few small-sized recrystallized β grains. These results can be attributed to the storage energy of the original billet with a high density of LAGBs (Figure 1) [29], which provided the driving force for recrystallization in the longer hold period (180 s). The recrystallized grains grew rapidly at a high temperature (1200 °C) and were accompanied by some small secondary recrystallized grains. In contrast, there was no recrystallization with holding times of 5 s or 60 s and there were a few initial recrystallized grains at 120 s. In each case, the lack of recrystallization was due to insufficient holding time. Moreover, the thickness of the Ι region increased with the extension of the holding time; the same was seen in the Ⅱ region for holding times from 5 s to 120 s.

**Figure 7:**
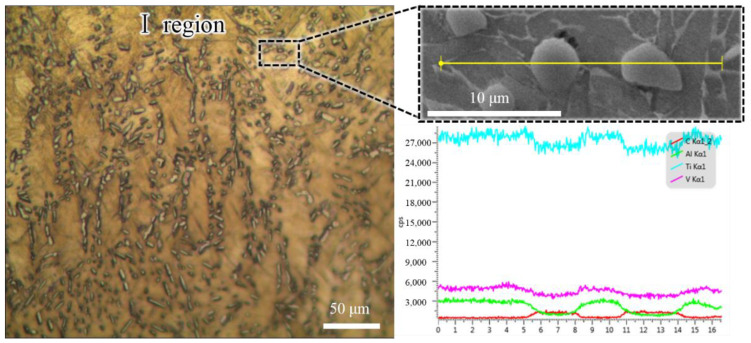
Metallographic and element distribution images of the Ι region.

As shown in Figure 7, the Ι region contained high-density particles with an average grains size of 3.06 μm. According to the line scanning results, these grains were rich in C and Ti elements. Upon further analysis using quantitative element detection by EMPA-WDS, the average content of C in these grains was 41.2%. Thus, these gains were TiC based on the equilibrium Ti-C phase diagram [30], and the C originated from the graphite punch.

**Figure 8:**
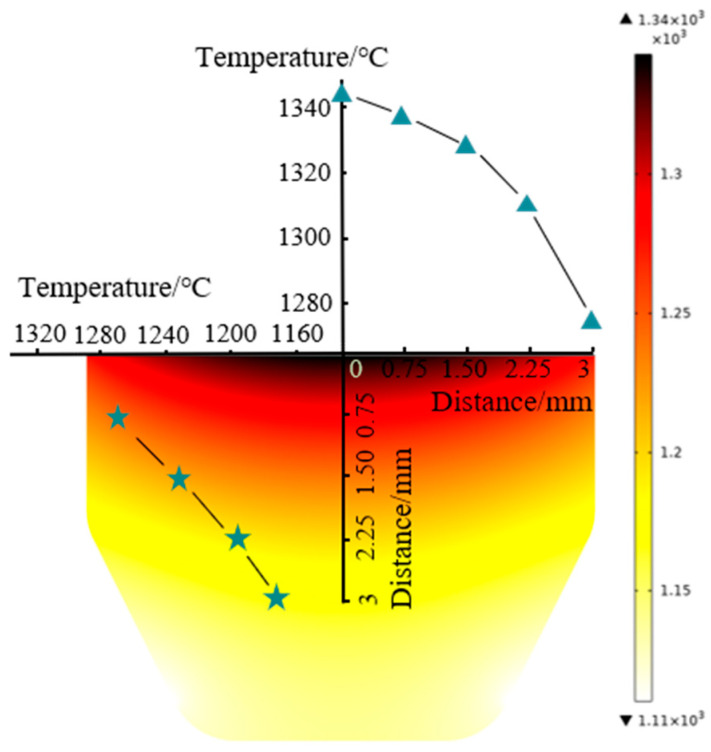
The temperature field distribution of the billet at the end of the holding time.

Figure 8 presents the billet’s temperature distribution at the end of the holding time. The temperature was generated by Joule heating. It was found that the temperature gradually decreased from the billet’s upper center to the sides. The billet exhibited a radial temperature gradient due to radiation from the outer graphite mold surface. At the billet’s upper surface, the center and edge temperature difference was 62 °C. Relevant literature [31] also revealed that the radial temperature gradient inside the TiN sample was 79 °C during the final dwell period at 1500 °C. Meanwhile, the longitudinal temperature gradient inside the billet was 230 °C higher than the radial temperature. The cause of the longitudinal temperature gradient has two aspects. On the one hand, due to the electrical current density, the billet’s upper surface was in contact with the graphite punch, while the lower end face was in a free state (Figure 2a). On the other hand, contact resistance between the billet’s upper surface and the graphite punch generated a large amount of Joule heat which transferred to the relatively low-temperature regions.

A temperature gradient distribution in the longitudinal and radial direction led to the inhomogeneous distribution of β grains and the formation of TiC layers. The TiC resulted from the carburizing between the graphite punch and the upper billet surface at elevated temperatures.

### 3.3. The Effect of TiC on the Extrusion Force

**Figure 9:**
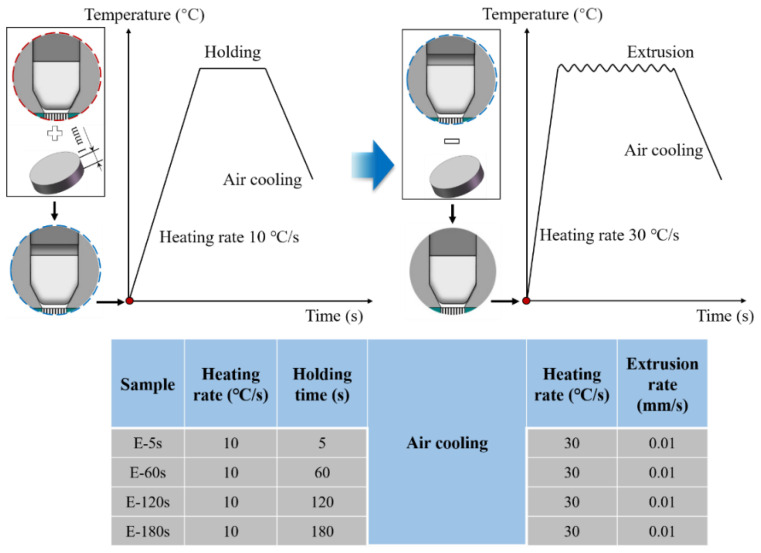
Schematic illustration of the extrusion processing routes for the same carburizing condition with different β grains.

As mentioned above, the longer the holding time, the greater the extrusion force. The inhomogeneous temperature distribution led to a carburized structure and gradient β grains inside the billets. In order to explore the influence of the carburized layer thickness and the β grain size on the extrusion force, as shown in Figure 9, a Ti-6Al-4V sheet with a thickness of 1 mm was added between the graphite punch and the billet. The temperature was raised to 1200 °C and was then held for different times. After air cooling and removal of the sheet, the graphite molds were heated to 1200 °C again at a rate of 30 °C/s, and they were then extruded. In this way, each billet sample had the original β grain distribution, as shown in Figure 6. The carburized layer only came from the process of elevating temperature and the thickness of the carburized layer was consistent.

**Figure 10:**
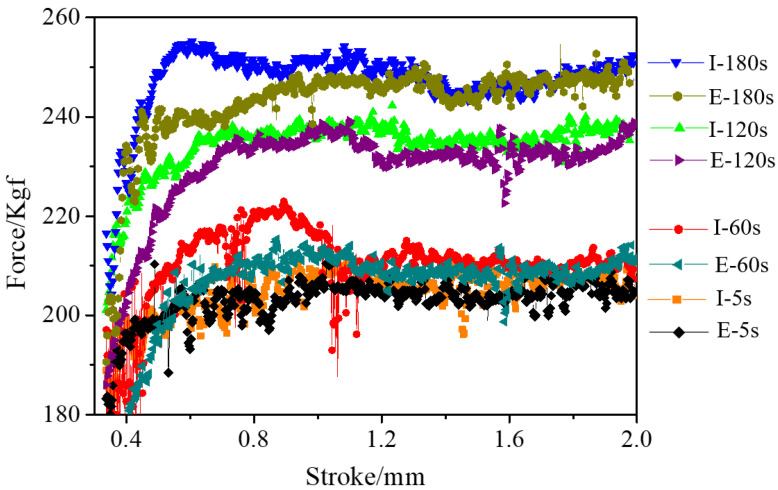
The extrusion force of different carburized layer thicknesses (I-5s, I-60s, I-120s, I-180s) and the extrusion force of carburized layers of the same thickness (E-5s, E-60s, E-120s, E-180s).

Figure 10 presents the extrusion force of the different thickness carburized layers (I-5s, I-60s, I-120s, and I-180s) and the extrusion force of the same thickness carburized layers (E-5s, E-60s, E-120s, and E-180s). The difference between I-5s and E-5s was minimal because of the short holding time. The extrusion force of I-60s and I-120s was slightly larger than that of E-60s and E-120s, respectively, indicating that the carburized layer impeded the plastic flow of the Ti-6Al-4V alloy at high temperature. However, although the thickness of the carburized layer of I-180s was the largest, there was no difference between I-180s and E-180s in terms of the extrusion force. This phenomenon demonstrated that the β grain size had a more prominent effect on the extrusion force than the carburized layer at the holding time of 180 s.

### 3.4. The Effect of β Grains on the Extrusion Force

**Figure 11:**
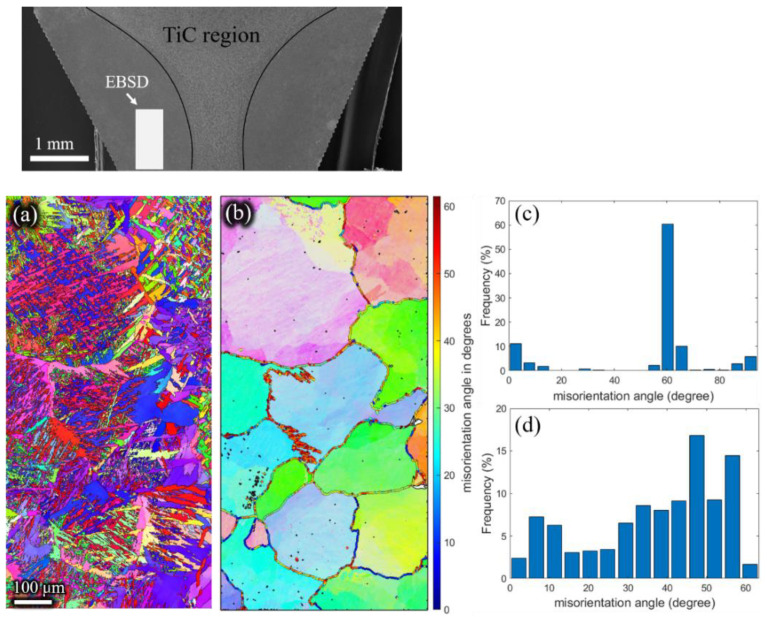
Inverse pole Figures of (**a**) the α phase, and (**b**) the reconstructed β phase. Misorientation angle of (**c**) α grains, and (**d**) reconstructed β grains of the residual deformed billet with a holding time of 5 s.

**Figure 12:**
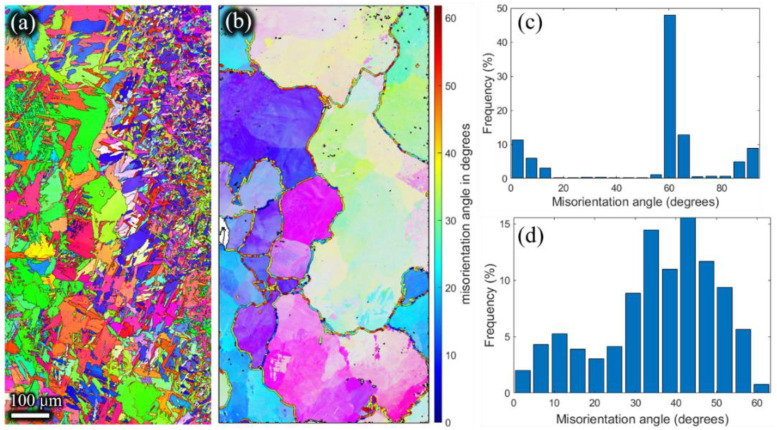
Inverse pole Figures of (**a**) the α phase, and (**b**) the reconstructed β phase. Misorientation angle of (**c**) α grains, and (**d**) reconstructed β grains of the residual deformed billet with a holding time of 60 s.

**Figure 13:**
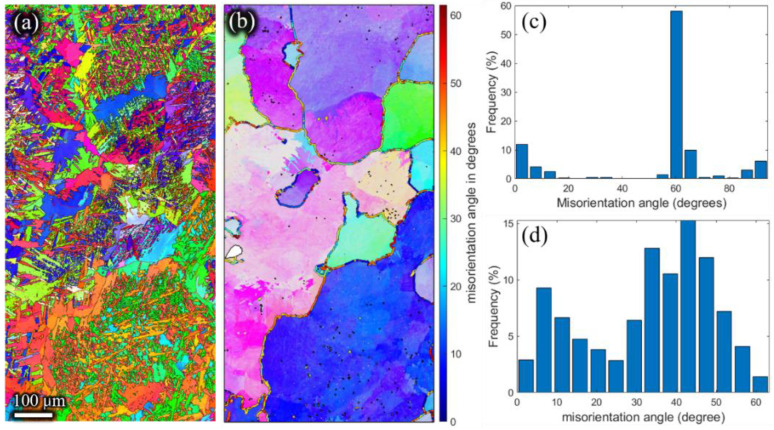
Inverse pole Figures of (**a**) the α phase, and (**b**) the reconstructed β phase. Misorientation angle of (**c**) α grains, and (**d**) reconstructed β grains of the residual deformed billet with a holding time of 120 s.

**Figure 14:**
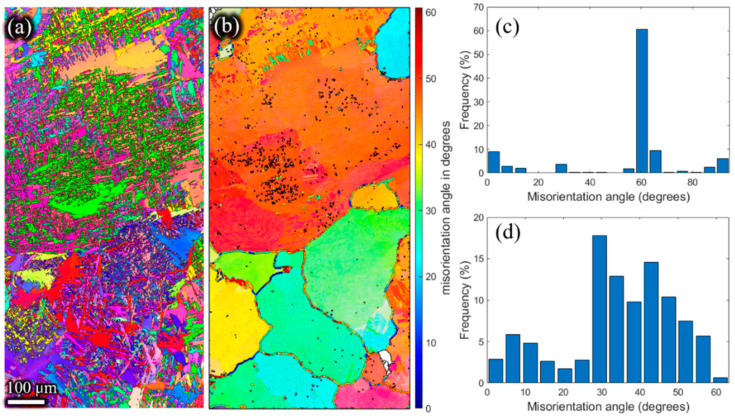
Inverse pole Figures of (**a**) the α phase, and (**b**) the reconstructed β phase. Misorientation angle of (**c**) α grains, and (**d**) reconstructed β grains of the residual deformed billet with a holding time of 180 s.

The inverse pole figures of the β phase (Figure 11b, Figure 12b, Figure 13b and Figure 14b) of the remaining billet after extrusion at different holding times were reconstructed on the basis of α inverse pole figures (Figure 11a, Figure 12a, Figure 13a and Figure 14a). The β grain size of the remaining billet increased with the prolongation of the holding time, which was mainly attributed to the correlation between the shorter the holding time, the less the storage energy consumption of the billet after the holding time, and the higher the degree of recrystallization in the subsequent extrusion process. However, there was no significant difference in β grain size at holding times of 120 s and 180 s (Figure 13b and Figure 14b), because prior coarse β grains were crushed in the hot extrusion process [32]. In addition, as indicated in Figure 6, the prior β grain size increased with the increase in the holding time. In conclusion, with the increase in the β grain size, the extrusion force increased. However, research literature [18,33] revealed that the flow stress has an apparent decreasing tendency with the coarsening of initial grains at ambient temperature because the decreasing grain boundaries facilitate the slip transfer among grains and further weakens the strength of the material and the resistance to deformation. In the present study, the β grain size increased while the grain boundary decreased according to the increase in the holding time. According to the studies [34,35], grain boundary sliding was a substantial contribution to high-temperature deformation of alloy, which was ascribed to the weaker strength of the grain boundary than the grain at high temperatures. Thus, the extrusion force increased with the extension of the holding time. In order to avoid brittle fracture and to reduce the abrasion of the graphite molds, the β grain size and the carburized layer thickness should be as small as possible by adjusting the holding time in the extrusion, which is conducive to reduced extrusion force.

Meanwhile, as shown in Figure 11d, Figure 12d, Figure 13d and Figure 14d, the high angle grain boundaries (HAGBs) of the reconstructed β grains accounted for about 80% of the residual deformed billet. Such a boundary angle distribution suggests that recrystallization occurred during the plastic deformation [35] since the dislocation accumulation and rearrangement formed subgrains in the deformation process. With increasing strain, these subgrains trapped more dislocations in the LAGBs which finally transformed into HAGBs, and thus new continuous dynamic recrystallization (CDRX) grains formed [36]. It can be inferred that the CDRX was the controlling mechanism during the plastic deformation of the micro-gears. When the high-temperature β phase transformed into the low-temperature α phase, the misorientation of α laths (Figure 11c, Figure 12c, Figure 13c,Figure 14c) was mainly concentrated at ~2°, ~10°, ~60°, and ~90°. The peak of ~2° was the existence of subgrain boundaries. Theoretically, the transformation of the prior β phase to the α phase should follow the Burgers orientation relationship. Therefore, each parent β grain can be converted into 12 different orientation variants of α. However, the misorientations showed only five distinct misorientation angle pairs because of crystal symmetry: 10.5°<0001>, 60°<11-20>, 60.8°<−1.377; −1; 2.377; 0.359>, 63.3°<−10; 5; 5; −3>, 90°<1; −2.38; 1.38; 0> [31], unlike the random distribution between the 12 α variants.

## 4. Conclusions

In this study, Ti-6Al-4V alloy micro-gears with a module of 0.3 mm and eight teeth, with a tooth thickness of 0.471 mm and a tooth depth of 0.675 mm, were manufactured at different holding times through hot extrusion processing. The extrusion force and microstructure evolution with the different holding times were studied based on the experiments and the simulations detailed in Section 2. The significant findings are summarized below.

(1) A gradient temperature distribution from the upper surface center to the periphery inside the billet was observed because of the contact resistance and electric current density. The temperature gradient resulted in a carburized layer and a gradient distribution of β grains.
(2) The carburized layer thickness and β grain size increased with the extension of the holding time. However, the β grain size was evenly distributed at a holding time of 180 s due to the rapid growth of recrystallized β grains.
(3) The extrusion force increased with the holding time because of the carburized layer thickness and the prior β grain size, while the β grain size was the dominant factor in the extrusion force. Therefore, a shorter holding time at high temperatures is conducive to the plastic forming of Ti alloys.
(4) The CDRX was the controlling mechanism during the plastic deformation of the micro-gears; the misorientation of the α variants followed the Burgers orientation relationship.

## Figures and Tables

**Table 1:** Manufacturing methods of micro-gears.

Reference	Material	Forming Method
[19]	Polymers	Micro-stereolithography
[20]	17-4PH stainless steel	Powder injection molding
[21]	Nickel	LIGA (lithography, electroplating, molding)
[22]	Polyurethane resins and green waxes	Vacuum-casting
[23]	SUS304	Stamping
[24]	7075 Al alloy	Extrusion
[25]	Steel and aluminum alloys	Forging
[26]	Beryllium copper	Wire electrical discharge machining

**Table 2:** Extrusion parameters for each holding time.

Sample	Heating Rate (°C/s)	Holding Time (s)	Extrusion Rate (mm/s)
I-5s	10	5	0.01
I-60s	10	60	0.01
I-120s	10	120	0.01
I-180s	10	180	0.01

## Data Availability

All data presented in this study are available from the corresponding author upon reasonable request.
